# Post-operative peritonitis due to a KPC-producing *Klebsiella pneumoniae*: the canvas of antibiotic resistance

**DOI:** 10.11604/pamj.2018.30.129.15532

**Published:** 2018-06-13

**Authors:** Marianneta Chatzopoulou, Maria Tsiakalou

**Affiliations:** 1 General Hospital of Larissa, Medical Microbiology, Larissa, 41221, Greece

**Keywords:** Multidrug-resistance, peritoneal fluid smear, klebsiella pneumoniae

## Image in medicine

Emergence of multidrug-resistant nosocomial pathogens has risen as one of the most critical public health issues globally. The present smear of peritoneal fluid graphically illustrates the dramatic course of an ultimately fatal case of post-operative peritonitis due to a KPC-producing *K. pneumoniae*. A 60-year-old patient was admitted for a scheduled sigmoidectomy. After a brief stay in the Intensive Care Unit he was transferred to the ward where he showed signs and symptoms of peritonitis. A peritoneal fluid film was stained with May-Grünwald-Giemsa technique and examined microscopically in the laboratory. The intensity of infection is reflected by the abundance of bacilli surrounding and invading neutrophils. The particular *K. pneumoniae* strain was merely susceptible to tigecycline and colistin. Aggressive antibiotic treatment was not effective in reversing the fulminant clinical course and the patient finally succumbed.

**Figure 1:**
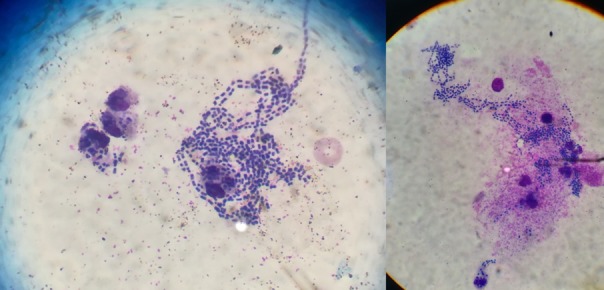
Peritoneal fluid smear stained with May-Grünwald-Giemsa technique and examined with 100x oil immersion objective; the film reveals abundant bacilli in serpentine configurations surrounding and invading host immune cells

